# FRCNet: Feature Refining and Context-Guided Network for Efficient Polyp Segmentation

**DOI:** 10.3389/fbioe.2022.799541

**Published:** 2022-06-29

**Authors:** Liantao Shi, Yufeng Wang, Zhengguo Li, Wen Qiumiao

**Affiliations:** ^1^ School of Automobile and Transportation Engineering, Shenzhen Polytechnic, Shenzhen, China; ^2^ School of Electronic and Information Engineering, University of Science and Technology Liaoning, Anshan, China; ^3^ Department of Mathematics, School of Sciences, Zhejiang Sci-Tech University, Hangzhou, China

**Keywords:** deep learning, polyp segmentation, enhanced context-calibrated module, progressive context-aware fusion module, multi-scale pyramid aggregation

## Abstract

Colorectal cancer, also known as rectal cancer, is one of the most common forms of cancer, and it can be completely cured with early diagnosis. The most effective and objective method of screening and diagnosis is colonoscopy. Polyp segmentation plays a crucial role in the diagnosis and treatment of diseases related to the digestive system, providing doctors with detailed auxiliary boundary information during clinical analysis. To this end, we propose a novel light-weight feature refining and context-guided network (*FRCNet*) for real-time polyp segmentation. In this method, we first employed the enhanced context-calibrated module to extract the most discriminative features by developing long-range spatial dependence through a context-calibrated operation. This operation is helpful to alleviate the interference of background noise and effectively distinguish the target polyps from the background. Furthermore, we designed the progressive context-aware fusion module to dynamically capture multi-scale polyps by collecting multi-range context information. Finally, the multi-scale pyramid aggregation module was used to learn more representative features, and these features were fused to refine the segmented results. Extensive experiments on the Kvasir, ClinicDB, ColonDB, ETIS, and Endoscene datasets demonstrated the effectiveness of the proposed model. Specifically, *FRCNet* achieves an mIoU of 84.9% and mDice score of 91.5% on the Kvasir dataset with a model size of only 0.78 M parameters, outperforming state-of-the-art methods. Models and codes are available at the footnote.^1^

## 1 Introduction

Colorectal cancer (CRC) is an ordinary malignant tumor of the gastrointestinal tract and is one of the most common types of cancer. Fortunately, CRC mortality can be greatly reduced if colon polyps, the bulging masses on the surface of the colon, are removed before CRC is formed (Kolligs, 2016). The localization and delineation of colon polyps play an important role in surgical treatment and medical care decision. Detailed boundary information can be provided by segmenting images of the polyp for subsequent clinical diagnosis and treatment. Several studies (Leufkens et al., 2012; Tajbakhsh et al., 2015b) have shown that approximately a quarter of polyps are missed during colonoscopy, which may increase the rate of missed diagnoses of colorectal cancer. Furthermore, colonoscopy procedures require polyp segmentation algorithms to present the results in real time to doctors to assist them in making suitable judgments and responses. At present, the main research direction is polyp detection and polyp segmentation technology. However, there are serious problems in the inspection methods of colorectal polyps. Due to the low contrast between the foreground and background information in the gastrointestinal channel, the accuracy of polyp resection in the process of endoscopic surgery under the image-level detection method cannot be guaranteed. Semantic segmentation gives a pixel-level classification in an image, that is, it classifies the pixels into its corresponding classes, whereas object detection classifies the patches of an image into different object classes and creates a bounding box around that object. To this end, the former can extract more abundant semantics than the latter, which is conducive to distinguishing the polyp tissue from the background well, thereby improving the probability of polyps detected. On the other hand, detection and localization of polyps are usually critical during routine surveillance and to measure the polyp load of the patient at the end of the surveillance while pixel-wise segmentation becomes vital to automate the polyp boundary delineation during the surgical procedures or radio-frequency ablations. To sum up, we argue that it is necessary to employ segmentation-based approaches to support colonoscopy.

**FIGURE 1:**
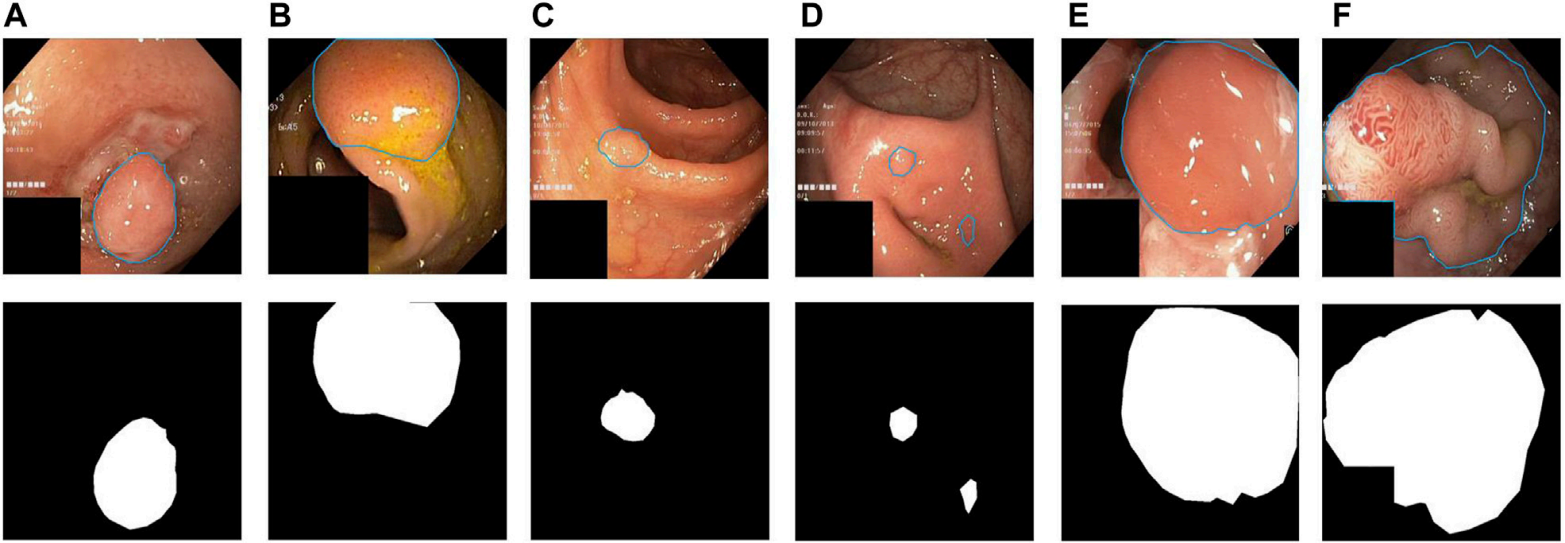
Typical challenging examples of polyp segmentation results: **(A,B)** the polyps with low contrast to the background, **(C,D)** the small polyps, and **(E,F)** the large polyps.

**FIGURE 2:**
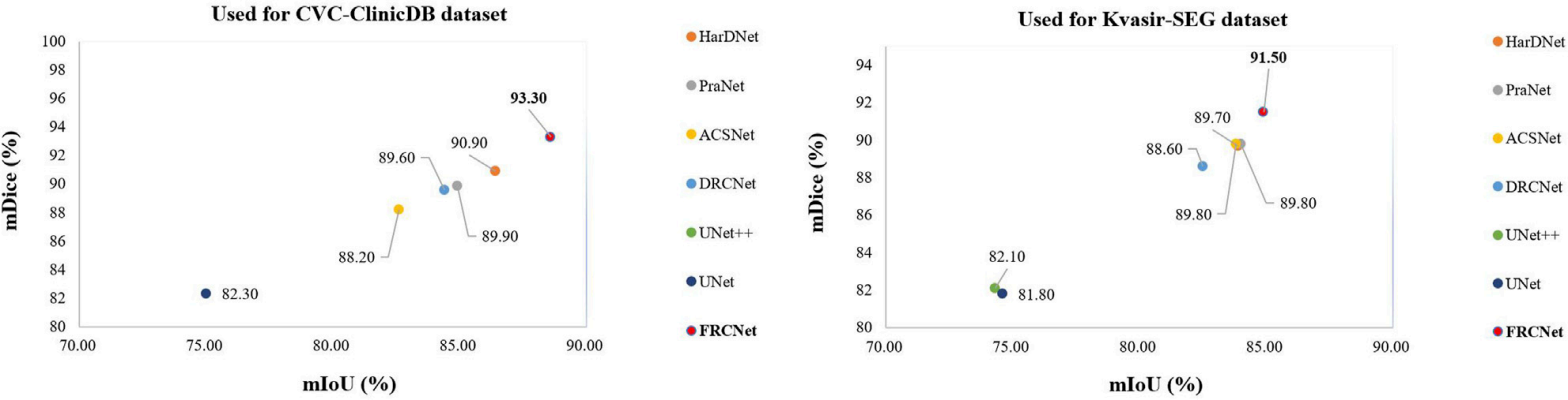
FRCNet shows better performance as compared to other state-of-the-art algorithms.

Precisely, segmenting polyps from colonoscopy videos is a challenging task. Firstly, the low contrast between the colon background and polyp foreground makes it difficult for the model to segment polyps from colonoscopy videos precisely, which may lead to false segmentation results of polyps (Figures 1A,B). Secondly, colon polyps can vary substantially in shape and scale (Figure 1C–F ). Thirdly, the segmentation results should be carried out in real-time so that the results can be presented to doctors immediately for prompt action during the colonoscopy. Figure 2 shows the network performance (Dice and IoU) of several current advanced algorithms on the Kvasir-SEG^2^ and CVC-ClinicDB datasets.^3^ As can be seen in the figure, the proposed FRCNet can achieve light-weight state-of-the-art performance.

It was difficult for early automatic polyp segmentation methods (Jerebko et al., 2003; Bernal et al., 2012; Ganz et al., 2012), to accurately separate the polyp target from the surrounding tissue because the polyps and surrounding mucosa have similar characteristics such as color, texture, scale and so on. Although some inherent characteristics of polyps can be utilized to distinguish a polyp from its surrounding background, such methods, which are based on hand-crafted features, are far from able to meet the above challenges. In recent decades, as deep learning and computer vision techniques have gradually developed and attracted researcher’s interest, a series of methods based on convolutional neural networks (CNNs) have been designed to segment polyps, substantially improving the accuracy of the segmentation results. For instance (Akbari et al., 2018), an image patch selection method based on a fully convolutional network (FCN) (Long et al., 2015) was proposed to perform polyp segmentation. However, owing to the inherent limitations of the FCN architecture, valuable detailed polyp boundary information may be lost after it has passed through a series of downsampling layers, which is fatal for pixel-level segmentation tasks such as semantic segmentation, especially medical image segmentation. In general, it is challenging to develop a method to meet the above-mentioned challenges of polyp segmentation and produce satisfactory results while maintaining real-time performance.

In order to yield satisfactory segmentation results meanwhile maintain real-time performance, in this study, we propose the feature refining and context-guided network (FRCNet), which is an adaptive context network for efficient polyp segmentation. First, we employ the enhanced context calibrated (ECC) module to obtain the most discriminative features by dynamically developing long-range spatial dependence through context calibration. Next, to address the large variation in the scale and shape of polyps, the progressive context-aware fusion (PCF) module is used to extract multi-scale contextual information. Finally, the multi-scale pyramid aggregation (MPA) module is developed to dynamically fuse the representative features output from multiple levels for refining the polyp segmentation map. Our experiments demonstrate that the proposed FRCNet can achieve better results than the state-of-the-art algorithms at a satisfactory speed. We can summarize the contributions of this article as follows:

• Two novel context modules, the ECC and PCF modules, were developed to effectively extract the most discriminative features and multi-range context information, respectively
• To generate more refined segmented results, we designed the MPA module to adaptively aggregate the multi-level output features
• Extensive experiments show that the proposed *FRCNet* achieves better results than other state-of-the-art methods while maintaining real-time performance

## 2 Related Work

### 2.1 Polyp Segmentation

Most early studies on polyp segmentation tasks rely on various hand-crafted features. For instance, Näppi and Yoshida (2002) employed the gradient concentration to differentiate between a polyp and the background. The Radon transformation (Deans, 2007) and Canny edge detection algorithm (Canny, 1986) have been used to segment images of polyp candidates by Jerebko et al. (2003). Using a combination of fuzzy c-means clustering and two-dimensional knowledge-guided intensity adjustment, Yao et al. (2004) designed an automatic method for reducing false positive detections in polyp segmentation. Gross et al. (2009) used multi-scale filtering and edge enhancement techniques to locate polyps, whereas Hwang and Celebi (2010) used Gabor texture features to further improve polyp segmentation accuracy. In some specific cases, these approaches can obtain good results but hand-crafted features are insufficient when the characteristics of the image become complicated, and hence they are unable to handle complex cases.

Recently, an innovative network, the FCN (Long et al., 2015), has achieved impressive results in semantic segmentation. In contrast to the hand-crafted features extracted by the traditional methods, the features extracted by the deep-learning–based methods are more discriminative and hence yield more precise results. In addition, there are also some excellent models in the field of target detection. (Li et al., 2019; Jiang et al., 2021a; Jiang et al., 2021b) which provide some advanced and efficient models. Bai et al. (Bai et al., 2022) provide an improved model based on deep feature fusion, which provides a new idea for future model optimization. Huang et al. (Huang et al., 2022) utilize multi-scale feature fusion, which can effectively focus on smaller target features. Optimization from multiple perspectives based on split attention networks and feature pyramid networks by Hao et al. (Hao et al., 2022) also provides a new solution for subsequent research. During the same period, U-Net (Ronneberger et al., 2015), which not only captures rich context information but also enables precise localization, was also recently proposed and has been applied in the field of medical image segmentation. Subsequently, many variants based on U-Net have been developed for polyp image segmentation. For instance, Li et al. (Li et al., 2017) directly employed an end-to-end U-shape structure for segmenting colorectal polyps. To further enhance the ability of model feature extraction, ResU-Net++ (Jha et al., 2019) used an atrous spatial pyramid pooling module (Chen et al., 2017; Chen et al., 2018) to extract multi-scale context information. DRCNet (Qin et al., 2020) enables each pixel to associate global semantic information by modeling the association of internal and external contextual information. To overcome the fact that polyps at different scales depend on different local or global contextual information, ACSNet (Zhang et al., 2020) uses a method that can adaptively select the context. PraNet (Fan et al., 2020) used a parallel method to predict the fuzzy regions, and used the attention mechanism to recover the boundary and internal region of the polyp, so as to achieve more accurate segmentation results. In the latest research, an HarDNet (Huang et al., 2021) method based on a simplified coding and decoding architecture was proposed. HarDNet improves the segmentation accuracy of the network while maintaining fast inference. Despite their success, the above methods are incapable and effectively model global context information to handle the large variation in polyps, in real-time performance.

### 2.2 Context Modeling

Context modeling is crucial for computationally intensive prediction tasks such as semantic segmentation, especially medical image segmentation. Moreover, the receptive field in the network determines how much context information is used. To enlarge the receptive field of the network, Yu and Koltun (2015) first designed an atrous convolution to comprehensively collect multi-scale context. Subsequently, Chen et al. (2017) designed an atrous spatial pyramid pooling block by manually and empirically setting atrous rates to capture multi-context information. Considering the full use of contextual information, a pyramid pooling module (Zhao et al., 2017) was designed to make use of the global context. Using a self-calibrated operation, SCNet (Liu et al., 2020) explicitly expands the fields-of-view of a network by adaptively building long-distance spatial dependencies. Inspired by the above approach, in this study, we developed two context-related methods, the ECC and PCF modules, which effectively extract the most discriminative features and multi-range context information, respectively.

## 3 Method

**FIGURE 3:**
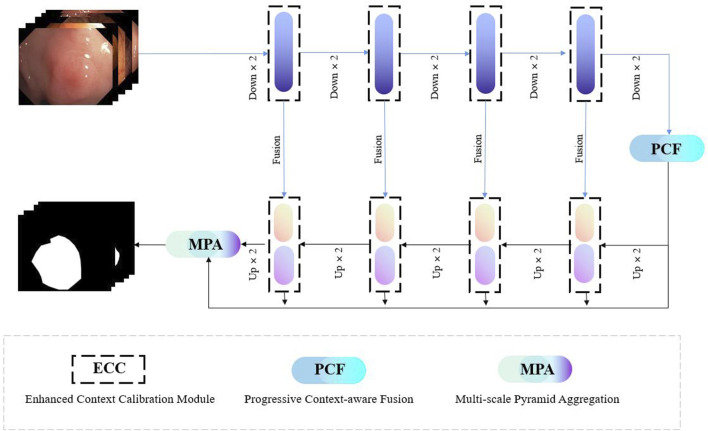
The overall architecture of our proposed FRCNet, which mainly includes three core modules, that is, ECC, PCF, and MPA. We employ the ECC and PCF module to encode more discriminative and multi-scale features, respectively. Finally, the MPA module is designed to adaptively fuse the output of the multi-level feature by the decoder for refining the final segmented result.

Our proposed FRCNet is depicted in Figure 3, where the overall architecture is based on the symmetrical classical encoder-decoder framework, which not only captures rich context but also enables precise localization. Due to the low contrast between the surrounding tissue and the polyps, we employ the enhanced calibration convolution (ECC) module to replace vanilla convolution and extract more discriminative features. At the bottom of the encoder, we further developed the progressive context-aware fusion (PCF) module that extracts multi-scale contextual information and can adapt to large variations in the scale and shape of polyps. Finally, to improve polyp segmentation accuracy in colonoscopy images, the multi-scale pyramid aggregation (MPA) module was designed and used in the decoder to learn more representative features by dynamically fusing the multi-level output features.

### 3.1 Enhanced Calibration Convolution Module

**FIGURE 4:**
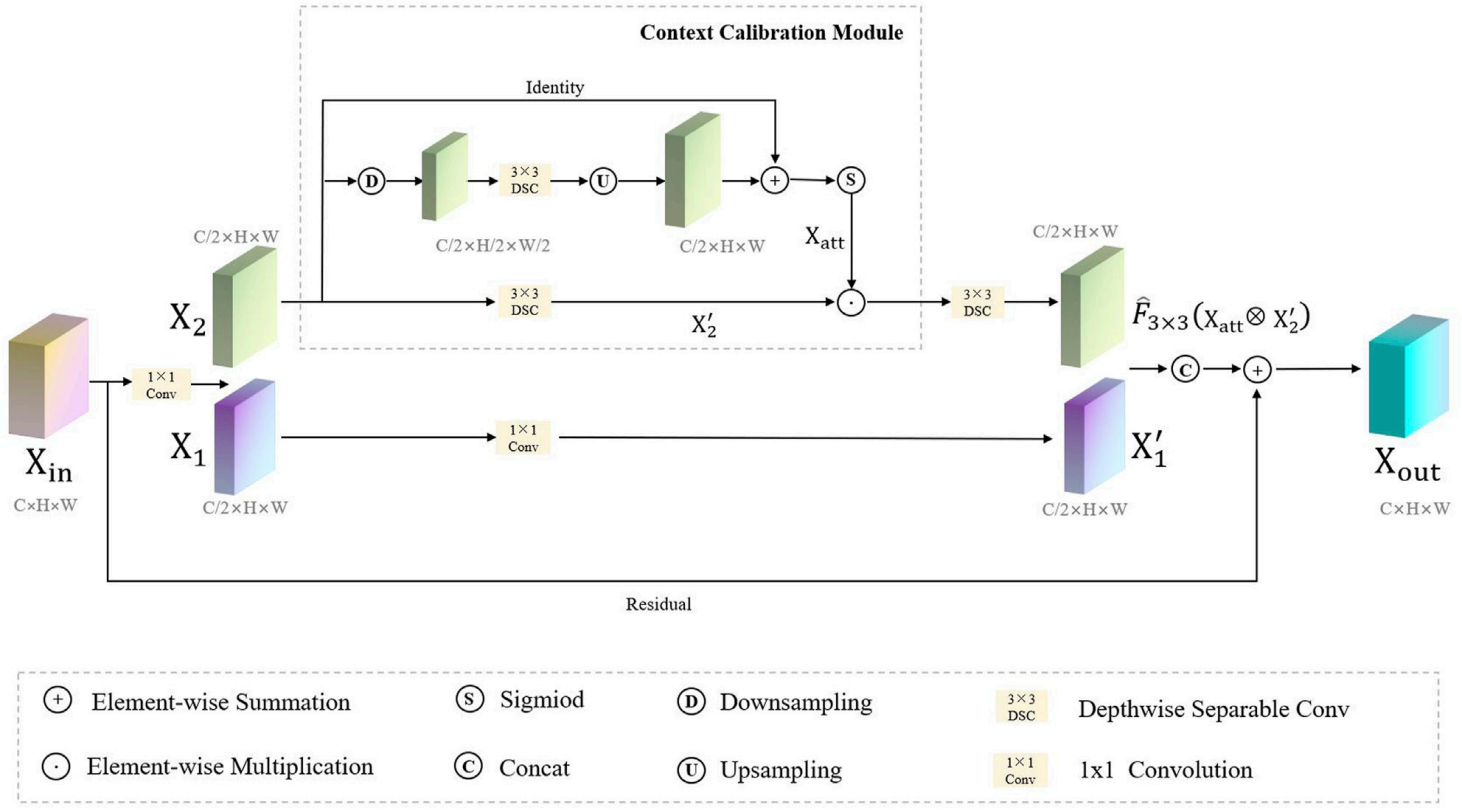
The flowchart of the ECC module.

Considering the trade-off between the computation and accuracy of the network, the size of the convolution kernel in traditional CNNs is usually fixed (e.g., 3 × 3). It can be seen that traditional convolutional neural networks usually use a fixed convolutional pattern to obtain a larger perceptual field by stacking the depth of the network, which will greatly limit the expressiveness of the network, resulting in important feature information that will be lost as the network deepens, thus making the model confusing and unable to distinguish polyps from the background tissue. Therefore, rather than introducing a more complex network architecture, we employ a context-calibrated operation to help the network learn more discriminative features. As Figure 4 shows, the ECC module is implemented *via* a split-fuse-select strategy.

In FRCNet, for any given input feature map $X_{\mathrm{in}}\in R^{C\times H\times W}$ , as the default, we first split the input feature map into two new feature maps $X_{1}\in R^{C/2\times H\times W}$ and $X_{2}\in R^{C/2\times H\times W}$ , according to the channel dimensions *via* a 1 × 1 convolution. Related research (Chen et al., 2019; Han et al., 2020) has shown that there are a substantial number of redundant feature maps in CNNs, which may reduce the feature extraction efficiency of CNN-based models. To address this problem, we first process **X**_**1**_ with a 1 × 1 convolution followed by a batch normalization algorithm and ReLU non-linear activation function, keeping the original feature transformation, as shown in the below part of Figure 4. Thus, the output feature map $X_{1}^{\prime }\in R^{C/2\times H\times W}$ can be generated. On the other hand, to reduce the interference of background tissues, the context-calibrated operation is specifically designed to develop long-range spatial dependence, described below.

As shown in the upper part Figure 4, we first perform the transformation $\hat{F}:X_{2}\to X_{2}^{\prime }\in R^{C/2\times H\times W}$ with a kernel size of three. Note that $\hat{F}$ is composed of convolution, batch normalization, and a ReLU activation function in that order. For further efficiency, depthwise separable convolution (Howard et al., 2017) is adopted, which substantially enhances efficiency without significantly reducing effectiveness, because this enables the model to learn richer feature representations with fewer parameters. Next, we utilize a context calibration operation to obtain the attention map representing the importance in each feature map. The calibration operation is formulated as follows:

$$X_{att}=\sigma \left(X_{2}⊕\left(\mathrm{Up}\left({\hat{F}}_{3\times 3}\left(\mathrm{Down}\left(X_{2}\right)\right)\right)\right)\right)$$ (1)

where *σ* and ⊕ represent the sigmoid function and element-wise summation operation, respectively. Here, *Up*(⋅) denotes a regular bilinear upsampling operation and *Down* (⋅) denotes the downsampling operation. Mathematically, the output of the ECC module $X_{\mathrm{out}}\in R^{C\times H\times W}$ can be defined as follows:

$$X_{\text{out }}=X_{\text{in }}⊕\mathrm{Cat}\left(X_{1}^{\prime },{\hat{F}}_{3\times 3}\left(X_{\text{att}}⊗X_{2}^{\prime }\right)\right)$$ (2)

where ⊗ and ⊕ are the element-wise multiplication and summation operation, respectively, and the *Cat*(⋅) represents a concatenation. Clearly, when compared with vanilla convolution, the ECC module can encode more accurate and discriminative features because it uses the context-calibrated operation. The ECC module, not only models the dependencies between channels through a simple scaling mechanism (downsampling and upsampling), enlarging the receptive-field of the network, but also considers the contextual information around each spatial position rather than taking the global contextual information into account.

### 3.2 Progressive Context-Aware Fusion Module

**FIGURE 5:**
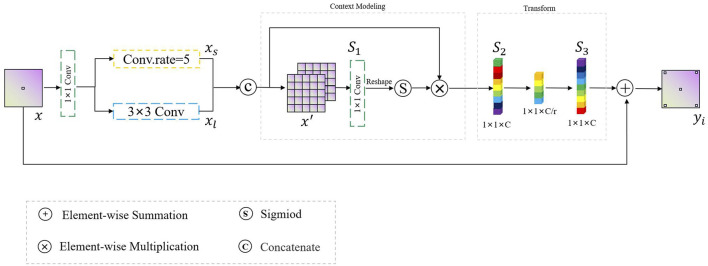
An overview of the proposed PCF module.

In this study, inspired by the global context block (Cao et al., 2019), we designed a PCF module to extract multi-range context information and guide the model to concentrate on the region of interest, in order to solve the problem of large shape changes in the process of polyp identification. The overall mechanism of the proposed PCF module is depicted in Figure 5. Features, also called as descriptors, the information extracted from images in terms of numerical values, are laborious to be perceived and correlated by humans. Surrounding features generally describe the image patches, whereas local features describe the smaller group of pixels. Intuitively, it is hard to understand the scene only depending on local features. Inspired by the human visual system, if we can obtain the region of interest and its surrounding contextual information, it will be easier to assign the category to the corresponding pixels.

Given a high-level abstract feature map $x\in R^{C\times H\times W}$ from the output of the encoder, we first feed it into a normal convolution to compress the channels of the network, reducing the complexity of the network. After that, we first used a 3 × 3 convolutional kernel for feature extraction, which is also called a local feature extractor **L** (⋅) due to the small receptive field obtained. Subsequently, to obtain richer contextual information, we used a dilated convolution with a dilation rate of 5 for feature extraction, which we refer to as a surrounding feature extractor **S**(⋅). The above two approaches extracted the feature maps $x_{l}\in R^{C/2\times H\times W}$ and $x_{s}\in R^{C/2\times H\times W}$ respectively. The surrounding context helps the network better distinguish the polyps from the background tissues. Intuitively, the recognition accuracy of the network can be further improved if we can consider the global context. To this end, the two feature maps **x**_**l**_ and **x**_**s**_ are concatenated as **x**′, where **x**′ = *Cat*(**x**_**l**_, **x**_**s**_). The next steps of context modeling and feature transformation are shown in Figure 5. First, we use 1 × 1 convolutions **S**_1_ to reshape the feature maps **x**′ and the softmax function to obtain the attention weights. Then, the global context is obtained by an attention operation. Second, the features are transformed *via* 1 × 1 convolutions **S**_2_ and **S**_3_. Finally, the channel-wise global context information is aggregated onto the channels of the original features. Thus, the output **y**_*i*_ of the PCF module can be expressed as follows:

$$y_{i}=x+S_{3}\mathrm{ReLU}\left(\mathrm{LN}\left(S_{2}\overset{T_{p}}{\underset{j=1}{\sum }}\beta_{j}x_{j}^{\prime }\right)\right)$$ (3)

Here, *T*_*p*_ = *H* ⋅ *W* is the number of locations in **x**′ and $\beta_{j}=\frac{e^{S_{1}x_{j}^{\prime }}}{\sum_{q}e^{S_{1}x_{q}^{\prime }}}$ is a weight for the global attention pooling for context modeling, and $\gamma \left(\cdot \right)=S_{3}\mathrm{ReLU}\left(\mathrm{LN}\left(S_{2}\left(\cdot \right)\right)\right)$ denotes the bottleneck transform for capturing channel-wise dependencies. Finally, we use a skip connection (He et al., 2016) for feature fusion to accelerate the network convergence. Therefore, compared with the input feature map x, in the output map y, the contextual information that exists in the target region has been strengthened.

### 3.3 Multi-Scale Pyramid Aggregation Moudle

In FRCNet encoding process, we jointly use the enhanced attention module ECC to extract features and the PCF module to suppress background noise in the high-dimensional semantic information. During the decoding process, the conventional convolution method is also replaced by the ECC module to recover the same resolution as the original input image. Although the inclusion of skip connection between the encoder and decoder is effective in bridging some of the spatially detailed information between the layers, the difference in semantic information between the layers still does not allow for more efficient information interaction. Since the foreground information of polyps in the intestine does not differ much from the background information and there is a lot of interference from background noise such as folds in the intestine, using only skip connection would lead to inaccurate polyp segmentation results. In order to solve the above problem, multi-level feature fusion strategies (Lin et al., 2017; Xie et al., 2020) were applied and were effectively proven to be capable of the above segmentation task. However, most previous studies (Zhang et al., 2018) have not taken into account the semantic information gap between different levels, and the traditional feature fusion approach only performs feature fusion by direct pixel-level summation, which inevitably leads to degradation of segmentation accuracy. It is widely accepted that deep networks have a powerful ability to express hierarchical features, with low-level features focusing on edge or texture information but lacking sufficient semantic information, and high-level features on the contrary. Therefore, combining high-dimensional and low-dimensional information through a dynamic modeling approach will greatly improve the accuracy of the model.

**FIGURE 6:**
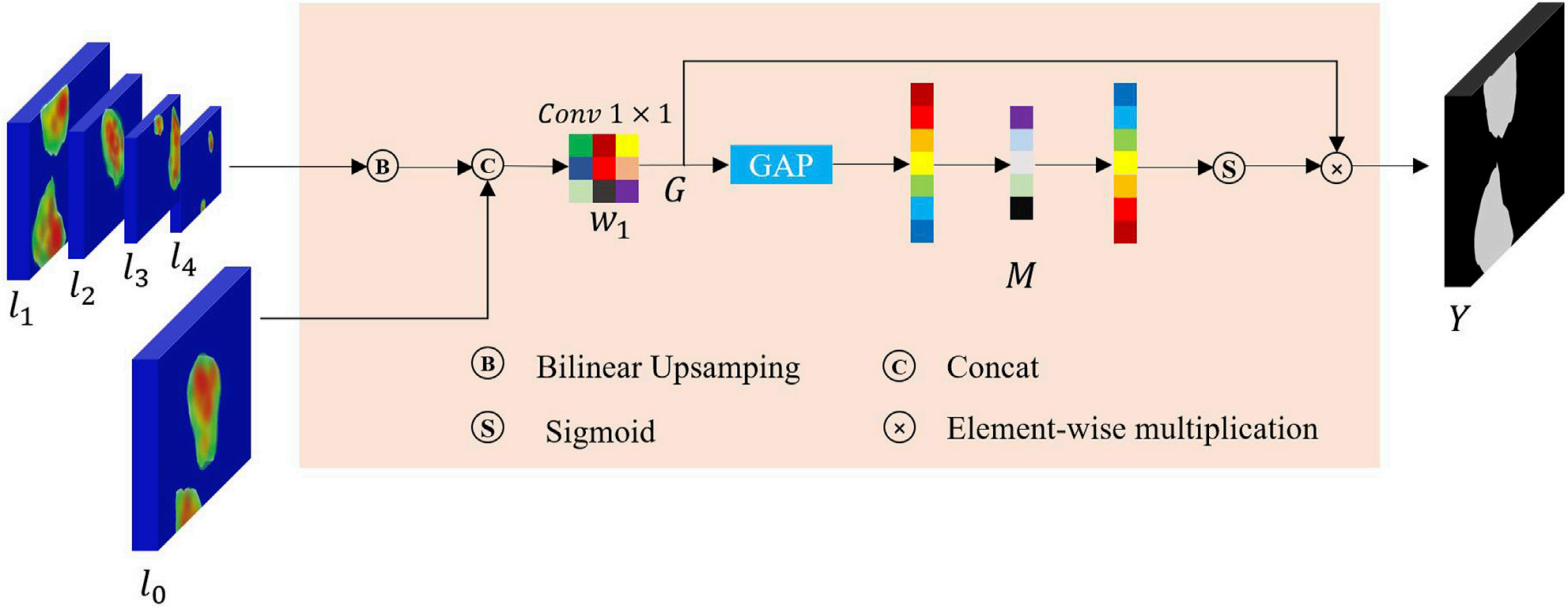
Illustration of the proposed MPA module.

To this end, in order to avoid performance penalty and to retain more fine-grained information to capture as many detailed features of the network as possible, a multi-scale pyramid aggregation (MPA) module was designed to collect more important details to refine the final segmentation results as shown in Figure 6. Finally, considering the disparity between different levels of output feature maps, we further employed SENet (Hu et al., 2018) to adaptively fuse multiple levels of output features, thus improving the overall fusion efficiency. Assuming that there exist **k** output layers of the decoder from the model given the multi-level output features $L=\left[l_{0},l_{1},l_{2},l_{3},l_{4}\right]\in R^{C\times H\times W}$ , we first perform a bilinear upsampling operation **B** to unify them to the same spatial resolution and then concatenated them to obtaining **G**, which is also fed into a feature projection function **W**_1_ to reduce the number of channel dimensions

$$G=W_{1}\left(\mathrm{Concat}\left(B\left(l_{4},l_{1},l_{2},l_{3}\right),l_{0}\right)\right).$$ (4)

Finally, a squeeze-and-excitation technique (Hu et al., 2018) is employed to re-weight the rough features and yield the final refined segmentation results:

$$Y=\left(G⊗\delta \left(M\left(g\left(G,\omega \right)\right)\right)\right),$$ (5)

where (ω) is the relative parameter. Function $g(\mathbf{X}) = \frac{1}{H \times W} $\sum_{i=1}^{H}\sum_{j=1}^{W}X\left(i,j\right)$ is calculated using global average pooling (GAP) to generate the channel-wise statistics. Moreover, **M**(⋅) represents the information interaction among channel dimensions. *δ*(⋅) denotes the sigmiod function, which is used to obtain the attention weight maps, and then pixel-level multiplication ⊗ is used to re-weight **G**.

### 3.4 Loss Function

Loss functions are one of the significant ingredients in colonoscopy image segmentation. Because there is a serious imbalance in the ratio of foreground (polyp regions) to background tissue in colonoscopy images, a comprehensive loss function is required to enable the network to converge faster and better. A significant advantage of the most commonly used loss function, binary cross-entropy loss, is that it can converge very quickly, but it is easily affected by any imbalance in the categories. It is expressed as follows:

$$L_{\text{BCE}}=-\underset{i}{\sum }\left(t_{i}\ln\left({\hat{t}}_{i}\right)+\left(1-t_{i}\right)\ln\left(1-{\hat{t}}_{i}\right)\right),$$ (6)

where *t* and $\hat{t}$ represent the polyp ground truth and polyp region predicted by the network, respectively. To handle the class imbalance problem, FRCNet also employs the Dice loss (Milletari et al., 2016), which is defined as follows:

$$L_{\text{Dice}}=1-\frac{2\cdot \left\langle t_{\left(h,w\right)},{\hat{t}}_{\left(h,w\right)}\right\rangle +\xi }{{\left\|t_{\left(h,w\right)}\right\|}_{1}+{\left\|{\hat{t}}_{\left(h,w\right)}\right\|}_{1}+\xi },$$ (7)

where (*h*, *w*) refers to the pixel coordinates and *ξ* is the Laplace smoothing factor to speed up the network convergence. Here, we set the *ξ* to 1e-8 in our works. Finally, by combining the abovementioned loss functions, we obtain the final loss function:

$$L_{\text{total }}=\lambda_{1}\cdot L_{\text{BCE}}+\lambda_{2}\cdot L_{\text{Dice}},$$ (8)

where *λ*_1_ and *λ*_2_ represent the relevant weight coefficient of the loss function. In this paper, we empirically set it to 0.6 and 0.4.

## 4 Experiments

### 4.1 Dataset and Evaluation

In this work, we conducted our experiments on five commonly used polyp datasets, including Kvasir-SEG (Pogorelov et al., 2017), CVC-ClinicDB (Bernal et al., 2015), CVC-ColonDB (Tajbakhsh et al., 2015a), EndoScene (Vázquez et al., 2017), and ETIS-Larib Polyp DB (Silva et al., 2014) datasets, to evaluate the effectiveness and efficiency of the proposed FRCNet. The Kvasir-SEG and ClinicDB datasets were our primary data sources for evaluating model learning ability. The Kvasir-SEG consists of 1000 labeled color polyp images that were captured from real colonoscopy video sequences, where the images vary from 487 × 332 to 1072 × 1920 pixels in size. Similarly, the images in the CVC-ClinicDB dataset were taken from the frames of 29 real colonoscopy videos. The dataset consists of 612 polyp images that are 384 × 288 pixels in size. We follow the setup in PraNet (Fan et al., 2020) and use 900 and 550 images from Kvasir-SEG and ClinicDB, respectively, as training sets, and keep 100 and 62 images, respectively, as test sets. In order to effectively test the generalization ability of the model, in addition to the Kvasir-SEG and ClinicDB datasets, we used three additional datasets for validation, ColonDB, ETIS, and EndoScene, which were not present during training and were open-sourced by different medical centers.

Several common metrics were adopted to quantitatively evaluate the FRCNet and other state-of-the-art methods. These metrics are mDice (Milletari et al., 2016), mIoU, mean absolute error (MAE), weighted F-measure $\left(F_{\beta }^{w}\right)$ (Margolin et al., 2014), S-measure $\left(S_{\alpha }\right)$ (Fan et al., 2017), and E-measure $\left(E_{\xi }\right)$ (Fan et al., 2018). Among these metrics, mDice and mIoU are similar in that they both indicate the degree of similarity at the region-level and focus on the consistency of the segmented objects within. MAE is a pixel-level comparison metric, and it is also capable of measuring the difference between predicted and labeled values. In order to solve the situation that precision and recall may contradict each other, we use F-measure $\left(F_{\beta }^{w}\right)$ to eliminate the contradiction. S-measure $\left(S_{\alpha }\right)$ , whose full name is Structure measure, is applied to measure the structural similarity between the original image and the image to be measured. E-measure $\left(E_{\xi }\right)$ is used to evaluate the segmentation results at pixel level and image level. In the results presentation section of this paper, we use *mE*_*ξ*_ and *maxE*_*ξ*_ to denote the mean and max value of the E-measure.

### 4.2 Implementation Details

FRCNet was implemented in the PyTorch framework on an Ubuntu 18.04.2 system in the Python 3.8 environment. During the training process, we adopted Adam optimization (Kingma and Ba, 2014) with a 1e-3 initial learning rate to optimize our model. In this work, we set the batch size to four and used an NVIDIA RTX 3090 Ti, which is a graphics card with 24 GB of G6X memory. To address the over-fitting problem and improve network performance, several data augmentation strategies were employed, including random horizontal and vertical flips, random rotations at 90° angles, and random adjustments to the brightness and contrast. The size of our model training input is 512 × 512 and our model is trained with at least 80 epochs to ensure full convergence. Note that, no image post-processing was needed in our study. Furthermore, all models were evaluated using the same experimental settings for fair comparison.

### 4.3 Ablation Studies

In this section, we present the results of several ablation studies to evaluate the major components in our proposed approach.

We conducted the ablation studies on five different datasets to evaluate the influence of different modules on our proposed FRCNet. In this ablation study, we used a U-Net-like architecture as our baseline model, where the output of each encoder layer is directly added instead of concatenated to the corresponding decoder layer for faster speed of inference. In the Baseline model, we next replaced the traditional convolutional operator with the ECC module to obtain Baseline + ECC, which has been proven to greatly reduce the number of redundant parameters. By further adding the PCF and the MPA module to the baseline model, we obtained another two models (Baseline + PCF and Baseline + MPA). Finally, we integrated the three modules into the Baseline model to obtain FRCNet.

**FIGURE 7:**
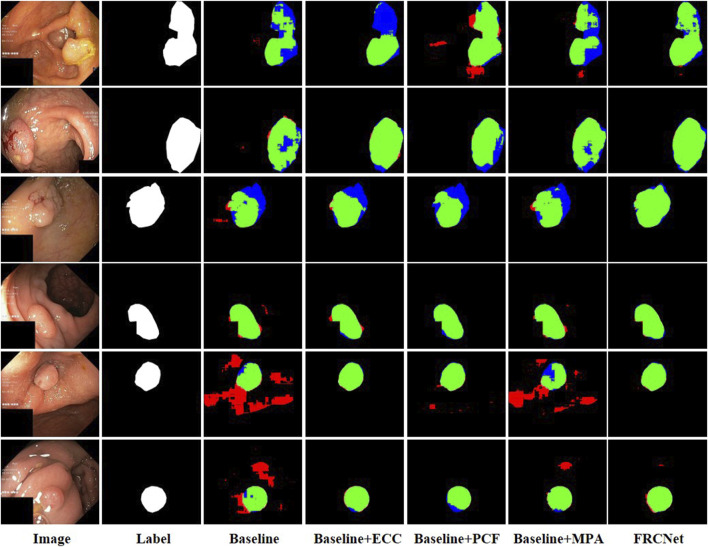
Visualization of the results of the ablation study.

We used 900 and 550 images from Kvasir-SEG and ClinicDB as training sets, and 100 and 62 images from Kvasir-SEG and ClinicDB as test sets, respectively, and we also used additional datasets from ColonDB, ETIS, and EndoScene to verify the generalization ability of the model, and, typical polyp segmentation results can be viewed in Figure 7. It is clear that the baseline method is unable to obtain acceptable segmentation results, especially under demanding conditions with extremely low contrast regions with irregular shapes and sizes. In comparison, by performing feature transformations in spaces with various field-of-view to collect more instructive contextual information for each object location, the Baseline + ECC method obtained more satisfactory results than Baseline, which can be observed that the background tissue area is suppressed well. Moreover, to address the varied irregular shapes and sizes challenges, the Baseline + PCF is capable of dynamically extracting multi-range context information for capturing the varied sizes and shapes of polyps by gradually combining local features, surrounding features, and global features, as can be seen in the fifth column of Figure 7. Furthermore, contributed by the effectiveness of the attention mechanism, multi-level output features can be adaptively fused after adding the MPA module to the Baseline, which could be refined as the final segmented results. As shown in the last column of Figure 7, the proposed FRCNet achieves the best performance, particularly on images with extremely low contrast or various polyp sizes and shapes. Furthermore, we also presented quantitative mIoU and mDice scores of the different models as shown in Table 1. The results show that directly replacing vanilla convolution with the ECC module, we can clearly observe that the Baseline + ECC model gains a higher score in the total analysis metrics. Adding the PCF module to the baseline, it improves nearly 10% over the baseline in mIoU and mDice, respectively, as shown in Table 1. The Baseline + MPA model also obtains better segmentation accuracy than Baseline, which indicates that the multi-level feature fusion is beneficial to boost performance. We have seamlessly integrated the above three modules together to form our FRCNet, which is nearly 15% ahead of the baseline on each dataset.

**TABLE 1:** Ablation studies of different modules on four different test datasets.

Settings	Kvasir		ClinicDB		ColonDB		ETIS		Endoscene	
	mDice	mIoU	mDice	mIoU	mDice	mIoU	mDice	mIoU	mDice	mIoU
Baseline	0.801	0.732	0.818	0.741	0.615	0.543	0.501	0.436	0.72	0.631
Baseline + ECC	0.889	0.823	0.901	0.862	0.711	0.64	0.663	0.61	0.857	0.782
Baseline + PCF	0.876	0.811	0.883	0.844	0.708	0.631	0.684	0.619	0.849	0.785
Baseline + MPA	0.856	0.798	0.861	0.805	0.687	0.613	0.591	0.531	0.823	0.712
**FRCNet**	**0.915**	**0.849**	**0.933**	**0.886**	**0.741**	**0.67**	**0.71**	**0.647**	**0.886**	**0.811**

The bolded value indicate that the obtained scores are the best and can be easily read by the reader.

### 4.4 Comparison With the State of the Art

To further evaluate the effectiveness and efficiency of FRCNet on polyp segmentation task, a comparison was made with several state-of-the-art algorithms: U-Net (Ronneberger et al., 2015), U-Net++(Zhou et al., 2018), DRCNet (Qin et al., 2020), ACSNet (Zhang et al., 2020), PraNet (Fan et al., 2020), and HarDNet (Huang et al., 2021). To make the comparison as fair as possible, we implemented all of the comparison methods and evaluated them on the five different datasets, including Kvasir-SEG (Jha et al., 2020), CVC-ClinicDB (Bernal et al., 2015), CVC-ColonDB (Tajbakhsh et al., 2015a), Endoscene (Vázquez et al., 2017), and ETIS-LaribPolypDB (Silva et al., 2014). The tested datasets use the same experimental settings, such as data augmentations methods and hardware environments.

**FIGURE 8:**
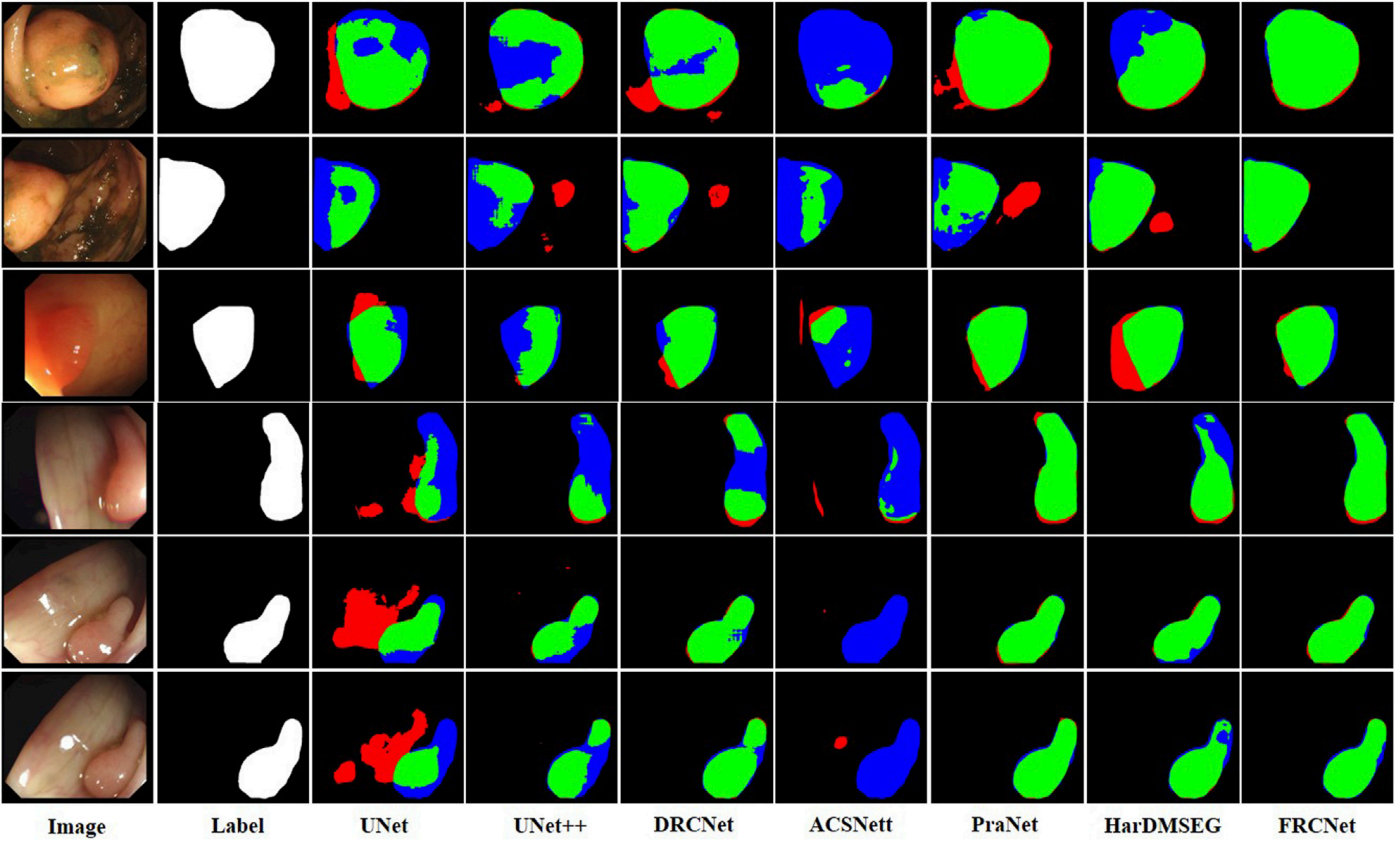
Visualization of the results of polyp segmentation on CVC-ColonDB dataset, where red, green, and blue colors in the figure are indicated as false positive, true positive, and false negative, respectively (best view in color).

**FIGURE 9:**
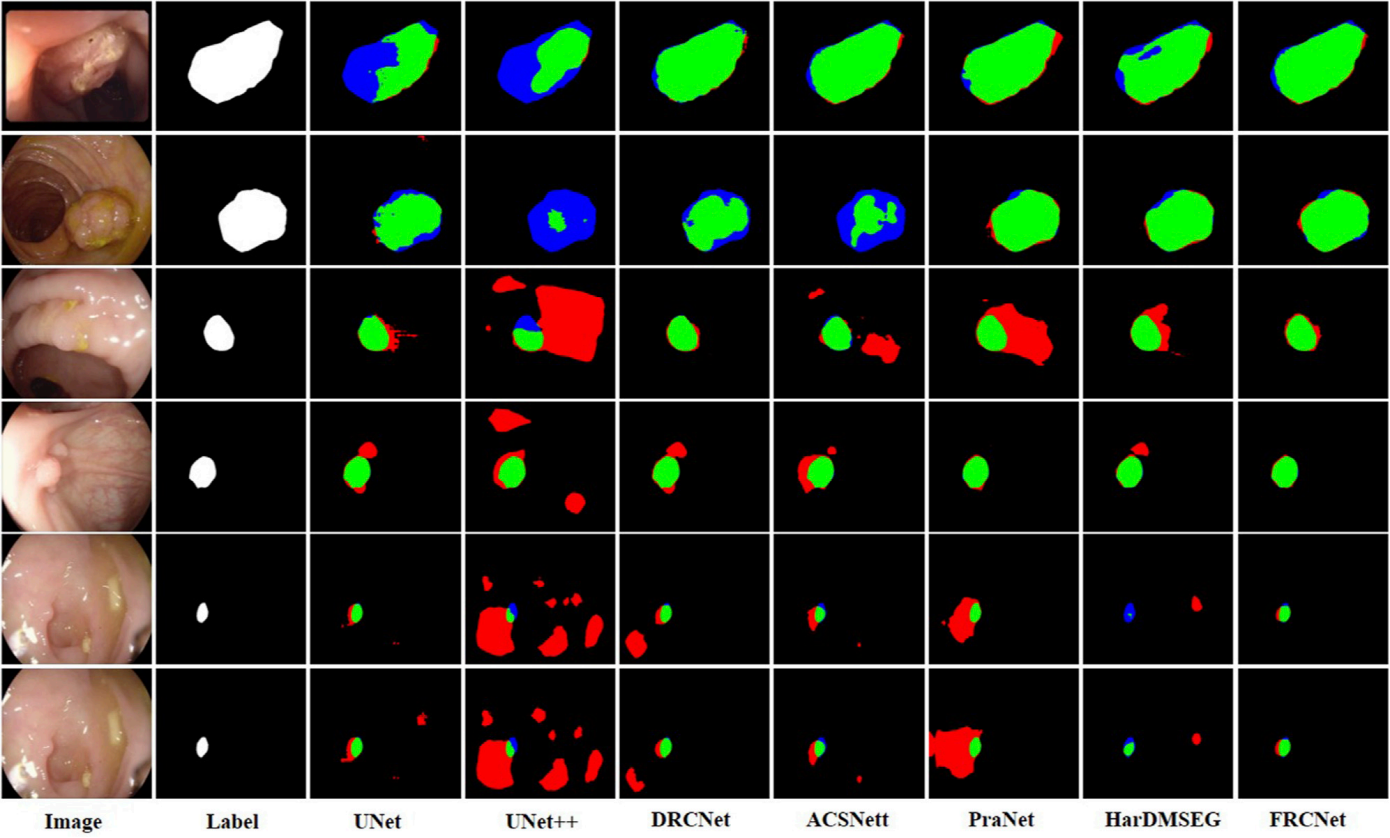
Visualization of the results of polyp segmentation on ETIS-LaribPolypDB dataset.

**FIGURE 10:**
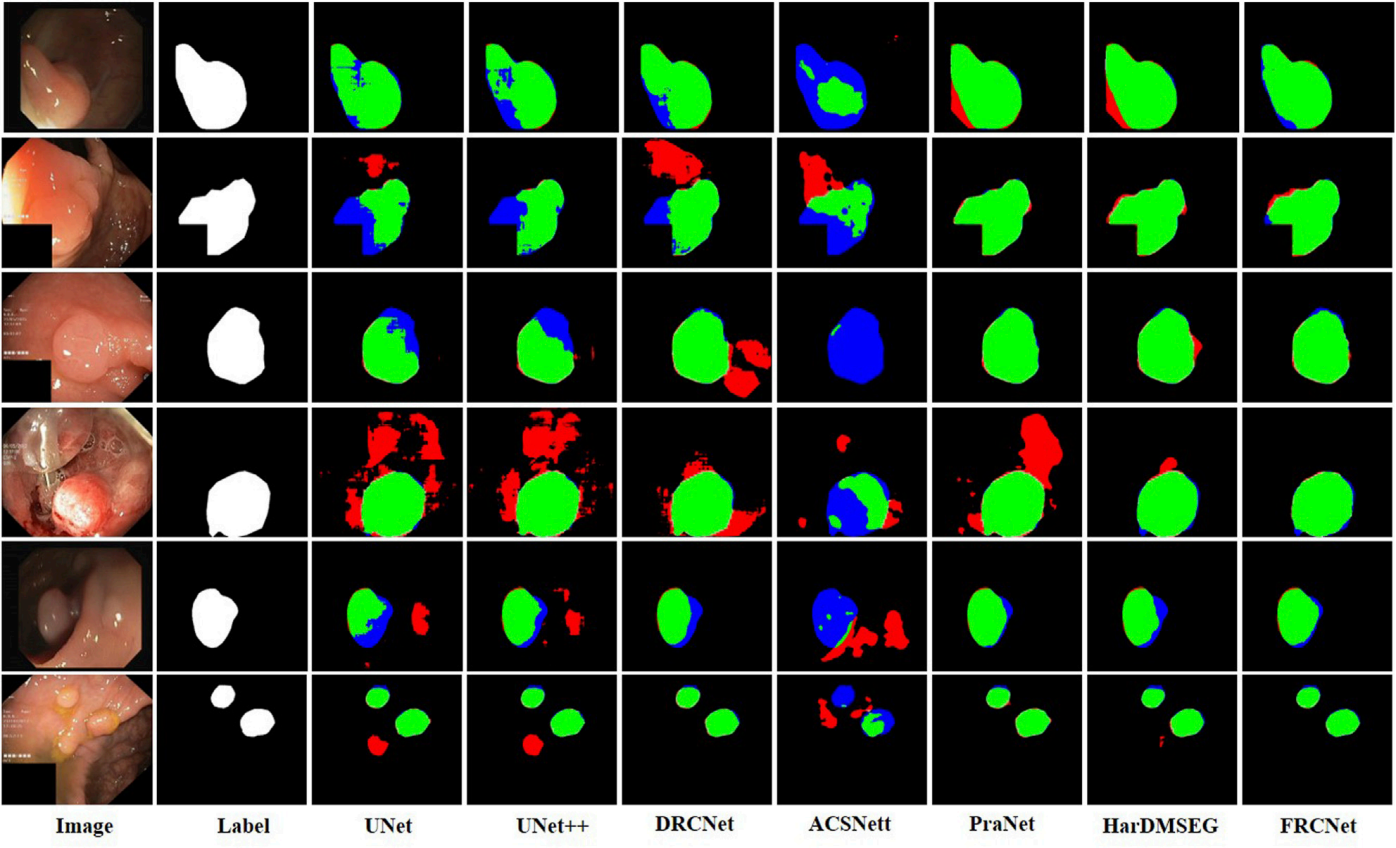
Visualization of the results of polyp segmentation on the Kvasir-SEG dataset.

The qualitative results show three sets of polyp segmentation result plots under different data sets, as shown in Figure 8, Figure 9, and Figure 10, which include numerous challenging cases with polyps of various sizes and irregular shapes. Moreover, the extremely low contrast between the foreground polyps and the background tissue may increase the probability of inaccurate segmentation. It is obvious to see that the classical U-Net is unable to handle the above challenging cases because of the limitations inherent in its architecture. U-Net++ outperforms the U-Net because it uses a residual technique to fuse the features effectively. DRCNet proposes a collaborative and interactive approach that uses internal and external contextual information to evaluate the similarity between each location of an image and all locations separately. As shown in the fourth column in Figure 9, UNet++ produces many false negative pixels because it does not have a sufficient global receptive field and context information. By contrast, ACSNet is able to adapt to more complex intestinal environments, and it enables the algorithm to maintain sensitivity to complex spatial environments, thus increasing the recognition accuracy of multi-scale polyps. Different from those UNet-based methods, PraNet is based on a parallel reverse attention mechanism, in which the reverse attention module is able to mine the cues of polyp boundaries and model the relationship between region and boundary information. Compared to previous competitors, HarDNet can produce more true positive pixels and achieve a satisfactory performance.

Despite their success, due to the inherent real-time requirements of the polyp segmentation task, the above algorithms do not meet the problem of clinical application. To comprehensively overcome the above challenges, by integrating three novel modules, that is, ECC, PCF, and MPA, the proposed FRCNet generally outperforms the other seven rivals. Compared to previous methods, the proposed FRCNet can not only effectively extract multi-scale features by fusing contextual information at a multi-range step by step, but also efficiently suppress the interference of background noise by building long-range dependence, to help the network learn more discriminative and useful features. Moreover, to gain a more refined dense prediction, based on attention mechanism and multi-level feature aggregation strategy, the MPA module is also developed to retain more representative features and more precise detailed information. Overall, the proposed FRCNet can not only segment polyps of a variety of large scales and irregular shapes but also effectively handle the complicated semantics variations of polyps.

In addition to the qualitative comparisons, we performed a statistical comparison to quantitatively evaluate the test results. As shown in Table 2, U-Net++ is slightly better than U-Net according to all estimate metrics. According to the table, the FRCNet achieved the highest mDice, reaching 0.915. By contrast, we can plainly discover that DRCNet, ACSNet, PraNet, and HarDMSEG all perform much better than the classical U-Net model with average improvements of 6%–8% in the mDice and mIoU. Furthermore, as the most competitive opponent, HarDNet achieves satisfactory performance with only 33.34 M parameters after the proposed FRCNet. Compared to the above-advanced algorithms, the proposed FRCNet achieves the highest performance in the vast majority of metrics, which demonstrates the effectiveness and efficiency of FRCNet. Note that, even given its remarkable performance, FRCNet only takes up 0.78 M parameters, indicating that it is suitable for use in colonoscopy procedures, which require fast polyp segmentation. A comparison of the performance on the CVC-ClinicDB, CVC-ColonDB, ETIS-LaribPolypDB, and EndoScene datasets are presented in Tables 3–6. The results reveal that FRCNet also achieved satisfactory performance on this dataset, again with lower computational complexity.

**TABLE 2:** Statistical comparison with different state-of-the-art methods based on the Kvasir-SEG dataset. The best results are bold faced.

Kvasir	mDice	mIoU	$F_{\beta }^{w}$	*S*_*α*_	*mE*_*ξ*_	*maxE*_*ξ*_	MAE	Param
U-Net	0.818	0.746	0.794	0.858	0.881	0.893	0.055	31.38
UNet++	0.821	0.743	0.808	0.862	0.886	0.91	0.048	9.16
DCRNet	0.886	0.825	0.868	0.911	0.933	0.941	0.035	-
ACSNet	0.898	0.838	0.882	0.92	0.941	0.952	0.032	-
PraNet	0.898	0.84	0.885	0.915	0.944	0.948	0.03	32.50
HarDMSEG	0.897	0.839	0.885	0.912	0.942	0.948	0.028	33.34
**FRCNet**	**0.915**	**0.849**	**0.911**	**0.919**	**0.948**	**0.959**	**0.024**	**0.78**

The bolded value indicate that the obtained scores are the best and can be easily read by the reader.

**TABLE 3:** Statistical comparison between different models on the CVC-ClinicDB dataset.

ClinicDB	mDice	mIoU	$F_{\beta }^{w}$	*S*_*α*_	*mE*_*ξ*_	*maxE*_*ξ*_	MAE
U-Net	0.823	0.75	0.811	0.889	0.913	0.954	0.019
UNet++	0.794	0.729	0.785	0.873	0.891	0.931	0.022
DCRNet	0.896	0.844	0.89	0.933	0.964	0.978	0.01
ACSNet	0.882	0.826	0.873	0.927	0.947	0.959	0.011
PraNet	0.899	0.849	0.896	0.936	0.963	0.979	0.009
HarDMSEG	0.909	0.864	0.907	0.938	0.961	0.969	**0.007**
**FRCNet**	**0.933**	**0.886**	**0.915**	**0.942**	**0.968**	**0.981**	**0.007**

The bolded value indicate that the obtained scores are the best and can be easily read by the reader.

**TABLE 4:** Statistical comparison between different models on the CVC-ColonDB dataset.

ClolonDB	mDice	mIoU	$F_{\beta }^{w}$	*S*_*α*_	*mE*_*ξ*_	*maxE*_*ξ*_	MAE
U-Net	0.512	0.444	0.498	0.712	0.696	0.776	0.061
UNet++	0.483	0.41	0.467	0.691	0.68	0.76	0.064
DCRNet	0.704	0.631	0.684	0.821	0.84	0.848	0.052
ACSNet	0.716	0.649	0.697	0.829	0.839	0.851	0.039
PraNet	0.712	0.64	0.699	0.82	0.847	0.72	0.043
HarDMSEG	0.735	0.666	0.724	**0.834**	0.859	0.875	0.038
**FRCNet**	**0.741**	**0.67**	**0.728**	0.831	**0.863**	**0.878**	**0.036**

The bolded value indicate that the obtained scores are the best and can be easily read by the reader.

**TABLE 5:** Statistical comparison between different models on the ETIS-LaribPolypDB dataset.

ETIS	mDice	mIoU	$F_{\beta }^{w}$	*S*_*α*_	*mE*_*ξ*_	*maxE*_*ξ*_	MAE
U-Net	0.398	0.335	0.366	0.684	0.643	0.74	0.036
UNet++	0.401	0.344	0.39	0.683	0.629	0.776	0.035
DCRNet	0.556	0.496	0.506	0.736	0.742	0.773	0.096
ACSNet	0.578	0.509	0.53	0.754	0.737	0.764	0.059
PraNet	0.628	0.567	0.6	0.794	0.808	0.841	0.031
HarDMSEG	0.7	0.63	0.671	0.828	0.854	0.89	**0.015**
**FRCNet**	**0.712**	**0.647**	**0.682**	**0.837**	**0.873**	**0.892**	0.036

The bolded value indicate that the obtained scores are the best and can be easily read by the reader.

**TABLE 6:** Statistical comparison between different models on the EndoScene dataset.

EndoScene	mDice	mIoU	$F_{\beta }^{w}$	*S*_*α*_	*mE*_*ξ*_	*maxE*_*ξ*_	MAE
U-Net	0.71	0.627	0.684	0.843	0.847	0.875	0.022
UNet++	0.707	0.624	0.687	0.839	0.834	0.898	0.018
DCRNet	0.856	0.788	0.83	0.921	0.943	0.96	0.01
ACSNet	0.863	0.787	0.825	0.923	0.939	0.968	0.013
PraNet	0.871	0.797	0.843	0.925	0.95	**0.972**	0.01
HarDMSEG	0.874	0.804	0.852	0.924	0.948	0.957	0.009
**FRCNet**	**0.886**	**0.811**	**0.853**	**0.927**	**0.956**	0.969	**0.008**

The bolded value indicate that the obtained scores are the best and can be easily read by the reader.

## 5 Conclusion

In this work, we presented a feature refining and context-guided network, called FRCNet, to comprehensively address the challenges of the polyp segmentation tasks. To suppress the background noise, we employed the ECC module to dynamically develop long-range spatial dependence while extracting the most discriminative features. Furthermore, to enable the network to segment polyps of different sizes and shapes, we proposed the PCF module, which adaptively captures multi-range context information. Finally, the MPA component was developed to learn more representative features for enhancing the final segmented results. Extensive experiments on five famous polyp datasets (Kvasir-SEG, CVC-ClinicDB, CVC-ColonDB, ETIS-LaribPolypDB, and EndoScene) demonstrate the advantages of the proposed FRCNet. Future investigations will include testing its robustness and generalization ability on more datasets and we believe that it could be easily extended to similar tasks in which varied sizes and shapes or ambiguous boundaries are the key challenges.

## Data Availability

The datasets presented in this study can be found in online repositories. The names of the repository/repositories and accession number(s) can be found at: https://datasets.simula.no/kvasir-seg/. https://polyp.grand-challenge.org/CVCClinicDB/.

## Footnotes

2
https://datasets.simula.no/kvasir-seg/

3
https://polyp.grand-challenge.org/CVCClinicDB/
